# Employing a Fuzzy-Based Grey Modeling Procedure to Forecast China’s Sulfur Dioxide Emissions

**DOI:** 10.3390/ijerph16142504

**Published:** 2019-07-13

**Authors:** Che-Jung Chang, Guiping Li, Shao-Qing Zhang, Kun-Peng Yu

**Affiliations:** 1 TSL Business School, Quanzhou Normal University, No. 398, Donghai Street, Quanzhou 362000, China; 2 Fujian University Engineering Research Center of Cloud Computing, Internet of Things and E-Commerce Intelligence, No. 398, Donghai Street, Quanzhou 362000, China; 3 Department of Management Science and Engineering, Business School, Ningbo University, No. 818, Fenghua Road, Ningbo 315211, China

**Keywords:** Grey system theory, small-data-set, forecasting, sulfur dioxide, emission

## Abstract

Effective determination of trends in sulfur dioxide emissions facilitates national efforts to draft an appropriate policy that aims to lower sulfur dioxide emissions, which is essential for reducing atmospheric pollution. However, to reflect the current situation, a favorable emission reduction policy should be based on updated information. Various forecasting methods have been developed, but their applications are often limited by insufficient data. Grey system theory is one potential approach for analyzing small data sets. In this study, an improved modeling procedure based on the grey system theory and the mega-trend-diffusion technique is proposed to forecast sulfur dioxide emissions in China. Compared with the results obtained by the support vector regression and the radial basis function network, the experimental results indicate that the proposed procedure can effectively handle forecasting problems involving small data sets. In addition, the forecast predicts a steady decline in China’s sulfur dioxide emissions. These findings can be used by the Chinese government to determine whether its current policy to reduce sulfur dioxide emissions is appropriate.

## 1. Introduction

As the most common oxy-sulfide, sulfur dioxide is a colorless gas emitting a strong pungent odor, and is a major pollutant in the atmosphere. Sulfur dioxide can cause respiratory tract inflammation, bronchitis, emphysema, conjunctivitis, and other health problems. It can also weaken the human immune system and reduce the ability to fight infections [1]. Industrial processes are among the major sources of sulfur dioxide emissions. Sulfur dioxide is generated during combustion during industrial processes, because coal and oil usually contain sulfur compounds. Sulfurous acid is formed when sulfur dioxide reacts with water. If sulfur dioxide is further oxidized, sulfuric acid (the main component of acid rain) is generally formed in the presence of a catalyst, such as nitrogen dioxide. This occurrence is one reason sulfur dioxide exerts a major impact on the ecosystem. In addition to its serious negative impacts on plants, animals, and buildings, sulfur dioxide directly leads to economic losses from metal corrosion. Sulfur dioxide emissions have to be strictly regulated because of their adverse impact on the environment, ecology, and economy [2]. To attenuate their impact on the ecosystem, significant efforts have been undertaken, and many countries are actively developing policies to reduce sulfur dioxide emissions [3,4,5]. The formulation of emission reduction policy is essential not only to improve living environments, but also to influence national industrial development. Reducing sulfur dioxide emissions requires long-term efforts to achieve an acceptable result, and inadequate policy direction could have a substantially negative impact.

Adequate emission forecasting is required to develop an effective policy to lower sulfur dioxide emissions, and can reduce errors in policy planning. Therefore, accurate predictions of sulfur dioxide emissions are essential for governments. Common forecasting methods are categorized into (i) the causal model, (ii) time-series analysis, and (iii) data mining methods. The causal model is used to explore the relationship between the independent variables and the dependent variables to forecast the corresponding values of the dependent variables [6]. Its forecasting performance depends on whether the selected independent variables can sufficiently explain the dependent variables. Time-series analysis considers the development of continuous data, and uses historical data to predict possible trends [7]. It has been widely used to solve forecasting problems; however, numerous observations are typically required for accurate predictions. Data mining involves searching for hidden information from collected data by using algorithms [8]. It can obtain satisfactory forecasts by adequate learning; however, the forecasting results depend on the amount of training data, and how effectively they represent the population. These limitations have yet to be overcome. 

For the aforementioned methods, the number of samples is the most vital factor that influences forecasting performance [7,8]. This characteristic renders these methods unsuitable for full application in various forecasting scenarios. One example is the forecasting problem on the sulfur dioxide emissions in China, where only a small amount of data on sulfur dioxide emissions have presently been collected [9]. Policies to reduce sulfur dioxide emissions must be drafted based on updated data. Using samples with updated information to construct a model can reflect the actual situation; thus, forecasting with a limited number of updated samples is valuable. Grey system theory was proposed by Deng (1982) [10] to handle uncertainty and insufficient information. Its main principle is to process data indirectly via accumulating generation operators (AGOs) to reveal regular patterns in data. Owing to simplicity and convenience, the method has been successfully applied in many fields [11,12,13,14]. The first-order one-variable grey model, abbreviated as the GM (1,1), is the main forecasting method in grey system theory; it requires only four observations to construct a model in order to obtain a satisfactory forecasting outcome, and can be used to address forecasting problems involving small-data-sets.

Studies indicate that grey models are effective analytical methods [15]. This study thus proposes an improved modeling procedure based on combining the grey system theory with the mega-trend-diffusion (MTD) technique [16]. It subsequently uses sulfur dioxide emission data from the environmental status report of the government of China to confirm the forecasting performance of the proposed method and its application value. The MTD is a virtual sample generation approach that has commonly been used to solve problems involving small-data-sets in various fields. The MTD is easy to construct, calculate, and use, and provides a feasible solution to practical problems. The present study applies the concept of MTD to develop an improved grey modeling procedure. In addition, a pre-test is performed before trend prediction to evaluate the use of the proposed technique. The experimental results demonstrate that the proposed method yields a satisfactory forecast with small data sets to solve encountered problems. Thus, it is considered a practical forecasting tool.

This study aims to develop a forecasting technique in order to determine future trends in sulfur dioxide emissions in China, and accordingly formulate relevant policies. The remainder of this paper is organized as follows: Section 2 introduces the proposed modeling method, Section 3 examines the forecasting performance of the adopted model and compares it with other prediction methods, Section 4 analyzes and discusses the results, and Section 5 presents the conclusions.

## 2. Methods 

The GM (1,1) has been widely applied, but its forecasting performance can still be improved. Chang et al. [17] suggested that adopting data-driven modeling based on the analysis of data characteristics could reflect data growth trends at different times. This concept could be applied to improve the performance of the conventional grey model. Accordingly, the current study applies the MTD to analyze the data characteristics, and subsequently propose a revised modeling procedure for solving the forecasting problem of sulfur dioxide emissions. Moreover, a rolling mechanism is adopted to enhance the medium-term forecasting ability of the proposed method. Specifically, the proposed modeling procedure mainly consists of three parts: the calculation of membership function value, grey modeling, and rolling mechanism. These parts are described in three subsections.

### 2.1. Mega-Trend-Diffusion Technique

The MTD technique, proposed by Li et al. in 2007 [16], is a method for solving modeling problems involving small data sets, and is often used to estimate the possible range of a data set. The main principle of the MTD technique is to fill data gaps between samples on the basis of data trends. The MTD assumes that samples should fall within a certain range, and that the likelihood of a single sample occurring can be conjectured using the fuzzy theory membership function (MF) [18]. In the MTD, the MF value represents the proximity of a single sample to a central location (CL), utilizing the importance of a single sample. The MTD technique can be used to analyze data behaviors and estimate the possible data profile under small data sets. These are the fundamental concepts used in the proposed approach to improve the forecasting accuracy of the grey model. The steps required to implement the MTD technique are summarized as follows:

Step 0: In a given data set X, let $\bar{e}$ be the element with the largest value and $\underline{e}$ be the element with the smallest value. 

Step 1: Calculate the CL using Equation (1).

(1) $$CL=\frac{\bar{e}+\underline{e}}{2},$$

Step 2: Determine the number of elements in the subset comprising data, with values greater than CL denoted as $N^{+}$. Determine the number of elements in the subset comprising data, with values smaller than CL denoted as $N^{-}$.

Step 3: Determine the positively and negatively skewed coefficients, $S_{U}$ and $S_{L}$, using Equation (2).

(2) $$\{\begin{array}{c} S_{U}=\frac{N^{+}}{N^{+}+N^{-}}\\ S_{L}=\frac{N^{-}}{N^{+}+N^{-}} \end{array},$$

Step 4: Calculate the variance of the sample using Equation (3).

(3) $${\hat{s}}_{x}^{2}=\frac{\sum_{i=1}^{n}{\left(x_{i}-\bar{x}\right)}^{2}}{n-1},$$

Step 5: Determine the estimated upper bound (UB) and lower bound (LB) using Equations (6) and (7). Equations (4) and (5) are the original settings of the boundary, but to avoid insufficient expansion in special cases, the formula is adjusted to Equations (6) and (7), where $\ln\left(10^{-20}\right)$ is the expansion coefficient.

(4) $$UB=CL+S_{U}\times \sqrt{-2\times {\hat{s}}_{x}^{2}/N^{+}\times \ln\left(10^{-20}\right)},$$

(5) $$LB=CL-S_{U}\times \sqrt{-2\times {\hat{s}}_{x}^{2}/N^{-}\times \ln\left(10^{-20}\right)},$$

(6) $$UB_{adj}=\{\begin{array}{ll} CL+S_{U}\times \sqrt{-2\times {\hat{s}}_{x}^{2}/N^{+}\times \ln\left(10^{-20}\right)} & UB\geq \bar{e}\\ \bar{e} & UB<\bar{e} \end{array},$$

(7) $$LB_{adj}=\{\begin{array}{ll} CL-S_{U}\times \sqrt{-2\times {\hat{s}}_{x}^{2}/N^{-}\times \ln\left(10^{-20}\right)} & LB\geq \underline{e}\\ \underline{e} & LB<\underline{e} \end{array},$$

**Figure 1:**
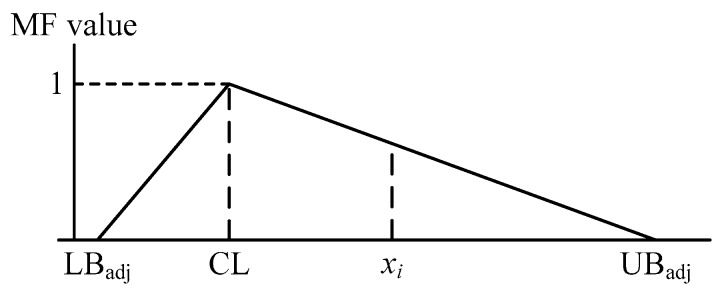
Triangular membership function (MF).

Step 6: Construct the triangular MF (Figure 1) and determine the MF values of the collected observations using Equation (8).

(8) $$MF=\{\begin{array}{cc} \left(x_{i}-LB_{adj}\right)/\left(CL-LB_{adj}\right) & x_{i}\leq CL\\ \left(UB_{adj}-x_{i}\right)/\left(UB_{adj}-CL\right) & x_{i}>CL \end{array},$$

### 2.2. Modeling Procedure

The background value of GM (1,1) significantly affects the forecasting performance of the grey model. Chang et al. [19] analyzed the role of the background value in the grey model. The background value possesses two main functions: (i) it alleviates the randomness by smoothing the data, and (ii) it emphasizes the importance of the newest datum. Consequently, a suitable setting for the background value should perform these two functions. Moreover, according to the fifth axiom of grey system theory (the principle of new information priority), the function of new pieces of information is greater than those of old pieces of information [20]. This indicates the importance of the newest datum when addressing the forecasting problems involving small data sets. The current study develops a formula for the background value to emphasize the importance of the newest data point in the model. The reconstructed calculation formula is based on the MTD technique, and allows the model to adapt to different data types. The proposed procedure is adopted to solve the forecasting problem for sulfur dioxide emissions.

**Figure 2:**
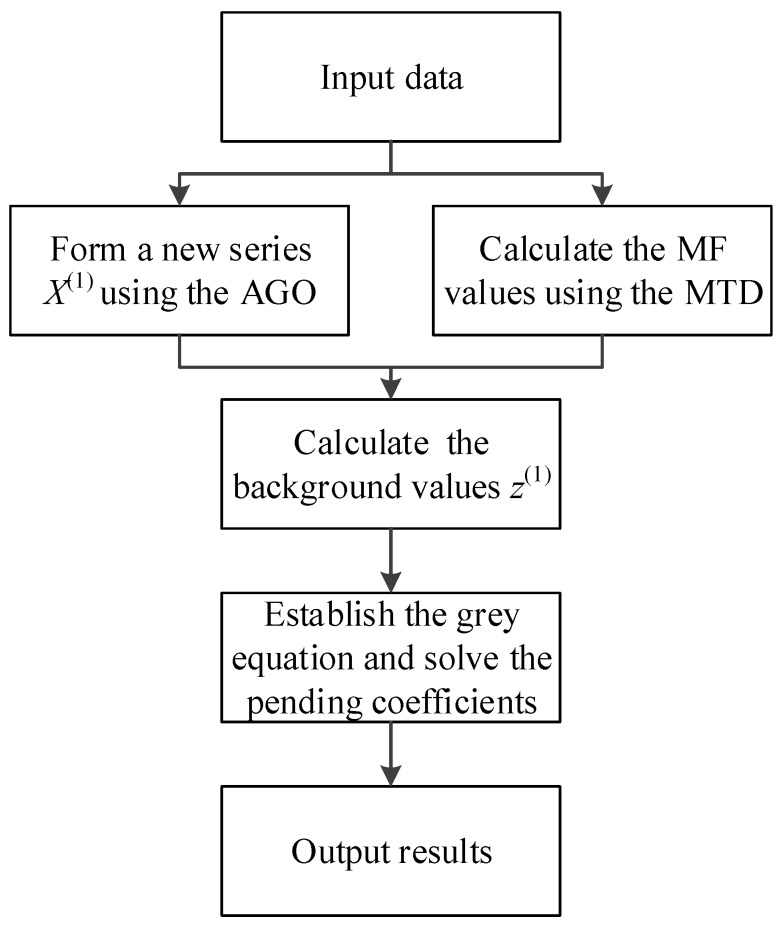
Flowchart of the proposed modeling procedure.

Figure 2 presents a flowchart of the proposed procedure, with its modeling steps briefly described as follows.

Step 0: Assume that *n* periods of non-negative time-series data are present, that is, $X^{\left(0\right)}=\left\{x^{\left(0\right)}\left(1\right),x^{\left(0\right)}\left(2\right),\ldots ,x^{\left(0\right)}\left(n\right)\right\}$.

Step 1: Calculate the MF values of the existing data using the MTD, $MF=\left\{MF_{1},MF_{2},\ldots ,MF_{n}\right\}$.

Step 2: Form a new data series using Equation (9), $X^{\left(1\right)}=\left\{x^{\left(1\right)}\left(1\right),x^{\left(1\right)}\left(2\right),\ldots ,x^{\left(1\right)}\left(n\right)\right\}$.

(9) $$x^{\left(1\right)}\left(k\right)=\sum_{i=1}^{k}x^{\left(0\right)}\left(i\right),\;k=1,2,\ldots ,n,$$

Step 3: Calculate the background value using Equation (10).

(10) $$z^{\left(1\right)}\left(k\right)=x^{\left(1\right)}\left(k-1\right)+MF_{k}\times x^{\left(0\right)}\left(k\right),\;k=2,3,\ldots ,n,$$

Step 4: Establish the grey differential equation and solve the pending coefficients of Equation (11) using the ordinary least squares method. Specifically, Equation (11) is expanded into Equations (13)–(16), and the coefficients are estimated from Equation (17). The original source equation, Equation (11), is replaced by Equation (12) to facilitate the calculation of the model.

(11) $$x^{\left(0\right)}\left(k\right)+az^{\left(1\right)}\left(k\right)=b,$$

(12) $$\frac{dx^{\left(1\right)}}{dt}+ax^{\left(1\right)}=b,$$

(13) $$\left[\begin{array}{c} x^{\left(0\right)}\left(2\right)\\ x^{\left(0\right)}\left(3\right)\\ \vdots \\ x^{\left(0\right)}\left(n\right) \end{array}\right]=\left[\begin{array}{cc} -z^{\left(1\right)}\left(2\right) & 1\\ -z^{\left(1\right)}\left(3\right) & 1\\ \vdots & \vdots \\ -z^{\left(1\right)}\left(n\right) & 1 \end{array}\right]\times \left[\begin{array}{c} a\\ b \end{array}\right],$$

(14) $$Y={\left[x^{\left(0\right)}\left(2\right),x^{\left(0\right)}\left(3\right),\ldots ,x^{\left(0\right)}\left(n\right)\right]}^{T},$$

(15) $$\hat{a}={\left[a,b\right]}^{T},$$

(16) $$B=\left[\begin{array}{cc} -z^{\left(1\right)}\left(2\right) & 1\\ -z^{\left(1\right)}\left(3\right) & 1\\ \vdots & \vdots \\ -z^{\left(1\right)}\left(n\right) & 1 \end{array}\right],$$

(17) $$\hat{a}={\left(B^{T}B\right)}^{-1}B^{T}Y,$$

Step 5: Solve Equation (12) together with the initial condition $x^{\left(0\right)}\left(1\right)=x^{\left(1\right)}\left(1\right)$, and obtain the desired forecast using Equations (18) and (19).

(18) $${\hat{x}}^{\left(1\right)}\left(k+1\right)=\left(x^{\left(0\right)}\left(1\right)-\frac{b}{a}\right)e^{-ak}+\frac{b}{a},$$

(19) $${\hat{x}}^{\left(0\right)}\left(k+1\right)={\hat{x}}^{\left(1\right)}\left(k+1\right)-{\hat{x}}^{\left(1\right)}\left(k\right),$$

### 2.3. Feasibility Assessment

Accuracy is a crucial index for evaluating the effectiveness of a forecasting model [21]; therefore, this study adopts the mean absolute percentage error (MAPE) to determine the forecasting performance in the pre-testing phase. MAPE is a relative percentage of errors that managers can used to assess the risks of adopting the forecasting tool [22]. The forecasting value and actual datum of the *i*th testing sample are represented by ${\hat{y}}_{i}$ and $y_{i}$, respectively. The MAPE is expressed as Equation (20).

(20) $$\mathrm{MAPE}=\frac{1}{m}\sum_{i=1}^{m}\left|\frac{{\hat{y}}_{i}-y_{i}}{y_{i}}\right|,$$

In the pre-testing phase, the forecasting results of the proposed procedure were compared of those obtained using two widely used forecasting techniques: the support vector regression (SVR; [23]) and the radial basis function network (RBFN; [24]), to confirm the feasibility of the proposed procedure. The SVR is a non-parametric estimation learning algorithm based on statistical learning theory to solve training problems with limited samples. It is one of the main methods used in machine learning because of its effective performance. The RBFN is an artificial neural network that adopts radial basis functions; it has multiple uses because of its convenience. Both the SVR and the RBFN were established using the machine learning software Weka 3.6.9 with default parameter settings.

### 2.4. Rolling Mechanism

Grey models exhibit adequate forecasting abilities; however, they are typically only suitable for short-term predictions and fail to capture medium-term trends [9,20]. To understand future trends in the coming years, the present study introduced the rolling mechanism into the modeling process in order to improve its medium-term forecasting performance. The rolling mechanism is a process by which data are metabolized. For instance, four given pieces of data, $\left\{x^{\left(0\right)}\left(1\right),x^{\left(0\right)}\left(2\right),x^{\left(0\right)}\left(3\right),x^{\left(0\right)}\left(4\right)\right\}$, are used to forecast the next output ${\hat{x}}^{\left(0\right)}\left(5\right)$ by using the proposed procedure. After the forecast is obtained, the newly forecast value is added to the data set, and the oldest datum $x^{\left(0\right)}\left(1\right)$ is removed from the data set to ensure the information is updated. Subsequently, the revised data set, $\left\{x^{\left(0\right)}\left(2\right),x^{\left(0\right)}\left(3\right),x^{\left(0\right)}\left(4\right),{\hat{x}}^{\left(0\right)}\left(5\right)\right\}$, is used to forecast the next output ${\hat{x}}^{\left(0\right)}\left(6\right)$. The process is repeated until all desired forecasting outputs are obtained.

The rolling mechanism can generally emphasize the immediateness of information, which is a common technique for time-series prediction. In addition, the forecasting errors of the grey model tend to deteriorate rapidly with the number of periods for multiple-step-ahead forecasting, because the grey model is based on an exponential function [20]. The rolling mechanism also provides a potential alternative to alleviate this occurrence. Prediction using the rolling mechanism may not be directly based on real data, but it possesses information to analyze future trends. Therefore, although not the optimal choice, the rolling mechanism is an acceptable expedient.

## 3. Results

The applicability of the proposed modeling procedure is assessed using a real case in the following subsections.

### 3.1. Data and Experimental Design

The experiment examined the effectiveness of the proposed modeling procedure for forecasting sulfur dioxide emissions. The study used the sulfur dioxide emissions data collected from the National Bureau of Statistics of China [25]. The data set contains nine-period annual observations ranging from 2007 to 2015 (Table 1). In the experiment, we used four data points to construct a model for predicting the next datum. That is, 2011’s forecasting value was inferred from the model based on the data from 2007 to 2010.

### 3.2. Modeling Example of the Proposed Procedure

The first four samples are used as examples to construct a forecasting model that explains the calculations of the proposed procedure. That is, a model based on the actual sulfur dioxide emissions in China from 2007 to 2010 was constructed to forecast the emissions in 2011. Specifically, {24.681,23.212,22.144,21.851} were used as inputs to create the grey model; a and b can be recognized as 0.03483 and 24.71380, respectively. Therefore, the forecasting model was ${\hat{x}}^{\left(1\right)}\left(k+1\right)=\left(-684.91656\right)e^{-0.03483k}+709.59756$, and the subsequent observation was predicted as $\hat{x}(5)\approx 21.118$.

### 3.3. Pre-Testing

The MAPEs in the proposed procedure, SVR, and RBFN are 2.96%, 3.83%, and 4.83%, respectively, as listed in Table 2. All values were less than 5% and fell within the level of highly accurate forecasting (Table 3; [26]), indicating that these techniques can be used to predict sulfur dioxide emissions in China. In addition, the proposed procedure exhibits higher forecasting accuracy, compared with the other two methods. These results reveal that the proposed procedure outperforms the other two methods in this case. Thus, the proposed method can be employed to solve the forecasting problems, given a limited number of samples, and is a suitable approach for forecasting short-term sulfur dioxide emissions.

## 4. Discussion

The pre-testing results and future trends of China’s sulfur dioxide emissions are explained in subsequent subsections.

### 4.1. Explanation of Pre-Testing Results

According to the comparison results in Table 2, the proposed procedure is superior to the other two methods; this implies that the proposed method can produce favorable results with small-data-sets. Overall, the pre-tested experimental results are consistent with general intuition and cognition: the grey-based approach can effectively handle prediction problems involving small data sets and provide good prediction accuracy; the development mechanism of SVR is not strict with the amount of data, and thus exhibits a high learning ability for limited samples; the performance of methods based on neural networks is determined by the sample size, and thus fails to obtain outstanding results. In addition, an appropriate increase in the number of samples may improve the forecasting performance of these three approaches. Lastly, all three methods yield highly accurate results and are thus suitable for short-term prediction of sulfur dioxide emissions in China.

### 4.2. Future Trends in Sulfur Dioxide Emissions in China 

**Figure 3:**
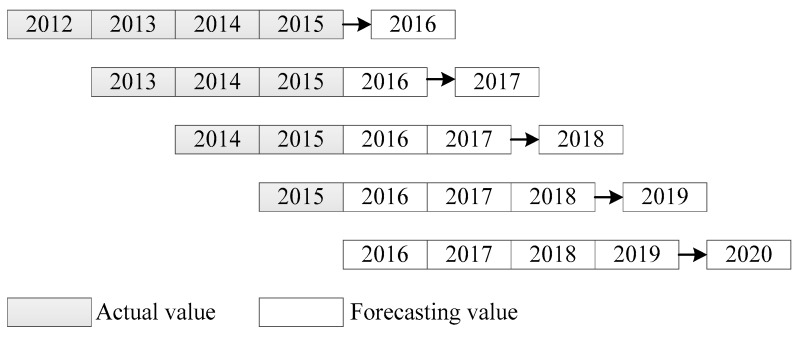
Schematic of the rolling mechanism.

To understand the future trends of sulfur dioxide emissions in China, this study integrated the rolling mechanism into the proposed modeling process to enhance its medium-term forecasting performance. A schematic of the rolling mechanism adopted in this study is presented in Figure 3.

The predicted values of sulfur dioxide emissions in China for the next five years, obtained using the proposed procedure with the rolling mechanism, are presented in Table 4. According to this forecast, sulfur dioxide emissions in China exhibit a steady decrease from 2016 to 2020. By 2020, sulfur dioxide emissions in China are expected to be 15.039 million tons, which reflects an improvement of approximately 20% relative to the emissions in 2015. The results indicate that policies reducing current sulfur dioxide emissions in China have been effectively implemented and have achieved positive results.

## 5. Conclusions

Effective forecasting of sulfur dioxide emissions is crucial in drafting policies to reduce sulfur dioxide emissions. However, such policies need to be drafted based on updated information. Therefore, the development of a suitable modeling procedure for forecasting using limited updated data is important.

Grey system theory can be used to construct forecasting models using small-data-sets, and can feasibly overcome the problem encountered in this study. This study provides an improved modeling procedure based on the grey system theory and the MTD technique in order to solve forecasting problems involving small-data-sets. The demonstration shows that the proposed method can yield satisfactory forecasts with a MAPE as low as 2.96%. This result indicates that grey system theory is practically useful when forecasting with limited data. Moreover, the results obtained using the SVR and the RBFN are not superior to those of the proposed model, which may be attributed to the small sample size. These two methods typically require a large training data set to obtain a robust model and prevent overfitting. Thus, SVR and RBFN’s forecasting performance could be improved if the training data sets were larger. Finally, the forecast indicates that sulfur dioxide emissions in China are steadily decreasing, suggesting the effectiveness of reduction policies to reduce sulfur dioxide emissions in China.

In future studies, the proposed method should be applied in other fields, such as engineering, industry, and energy to confirm its effectiveness. Combining a heuristic approach with the grey model to improve the accurate of the outcome would also be a valuable study direction. 

## Figures and Tables

**Table 1:** China’s sulfur dioxide emissions (unit: million tons).

Year	2007	2008	2009	2010	2011	2012	2013	2014	2015
Emissions	24.681	23.212	22.144	21.851	22.179	21.176	20.44	19.744	18.591

**Table 2:** Forecasting performances of various methods.

Methods	MAPE
Proposed procedure	2.96%
SVR	3.83%
RBFN	4.83%

**Table 3:** Mean absolute percentage error (MAPE) criteria.

MAPE	Forecasting Power
<10%	Highly accurate forecasting
10–20%	Good forecasting
20–50%	Reasonable forecasting
>50%	Inaccurate forecasting

**Table 4:** Forecast of China’s sulfur dioxide emissions (unit, million tons).

Year	2016	2017	2018	2019	2020
Forecasting value	17.956	17.074	16.397	15.680	15.039
